# MINARO HD: control and evaluation of a handheld, highly dynamic surgical robot

**DOI:** 10.1007/s11548-020-02306-9

**Published:** 2021-01-23

**Authors:** Manuel Vossel, Meiko Müller, Annegret Niesche, Lukas Theisgen, Klaus Radermacher, Matías de la Fuente

**Affiliations:** Chair of Medical Engineering, Helmholtz Institute for Biomedical Engineering, RWTH Aachen University, Pauwelsstraße 20, 52074 Aachen, Germany

**Keywords:** Surgical robotics, Handheld, Dynamic motion compensation, Real-time control, Tracking

## Abstract

**Purpose:**

Current surgical robotic systems are either large serial arms, resulting in higher risks due to their high inertia and no inherent limitations of the working space, or they are bone-mounted, adding substantial additional task steps to the surgical workflow. The robot presented in this paper has a handy and lightweight design and can be easily held by the surgeon. No rigid fixation to the bone or a cart is necessary. A high-speed tracking camera together with a fast control system ensures the accurate positioning of a burring tool.

**Methods:**

The capabilities of the robotic system to dynamically compensate for unintended motion, either of the robot itself or the patient, was evaluated. Therefore, the step response was analyzed as well as the capability to follow a moving target.

**Results:**

The step response show that the robot can compensate for undesired motions up to 12 Hz in any direction. While following a moving target, a maximum positioning error of 0.5 mm can be obtained with a target motion of up to 18 mm/s.

**Conclusion:**

The requirements regarding dynamic motion compensation, accuracy, and machining speed of unicompartmental knee arthroplasties, for which the robot was optimized, are achieved with the presented robotic system. In particular, the step response results show that the robot is able to compensate for human tremor.

## Introduction

Most current robotic systems for computer-assisted orthopedic surgery are large serial arms, comparable to anthropomorphic industrial robots. Examples of those systems are the ROBODOC [1], modiCAS [2], Mako [3], Mazor X [4] and ExcelsiusGPS [5]. They have to be slowed down during operation due to their large mass and the resulting inertia, as required by ISO 10218-1 and ISO/TS 15066. Nonetheless, this design induces specific risks due to a lack of inherent working space limits. [6] Another disadvantage of those systems is the large footprint of the robot’s base, i.e., a cart that is placed next to the operating table, in an already crowded operating room environment.

To overcome the issues of a large serial arm, small application-specific kinematics mounted directly on the patient’s bone, such as the MINARO [7], Arthrobot [8], MBARS [9] or Mazor Renaissance® (former SpineAssist) [10] were developed. Their main advantages are the partly inherent safety due to a limited workspace, low inertia and low spatial requirements [11]. Nevertheless, the rigid fixation to the patient’s bone required has a significant impact on the conventional surgical workflow (for fixation and, if necessary, sterile draping). One study found that surgeries with the Mazor Renaissance® took on average 30 min longer than freehand pedicle screw fixations [12]. Unicompartmental knee arthroplasty surgeries using the MAKO RIO system were found to take on average 27 min longer than using the Oxford approach [13].

Handheld robots have been proposed to combine the advantages of having a small robot system, being easy to handle in the intraoperative workflow without the need for a rigid fixation to the bone and with the benefit of versatile robotic tool guidance. The patient’s bone can also be tracked utilizing the tracking system used to localize the robot. Thereby, not only unintended motions induced by the operator can be compensated for but also movement of the bone due to forces applied by the surgeon or due to breathing [2, 14]. Examples are the intelligent tool drive for drilling [15] and the Micron for eye surgery [16].

Another type of handheld devices uses a position-dependent power or tool exposure control like the NAVIO surgical system [17]. Unlike the previously mentioned intelligent tool drive and Micron system, the NAVIO does not autonomously position the tool, but rather retracts the burr into a shaft (1 Degree of Freedom (DOF)) when the surgeon is going to leave the preplanned bone volume. On the one hand, this gives more freedom to the surgeon about the order in which to remove the bone. On the other hand, it is not possible to optimize the bone milling parameters such as milling speed, temperature rise and milling forces [18].

A new handheld, highly dynamic mini-robot (MINARO HD) was developed for burring applications based on the original MINARO kinematic structure [7]. Three actuators are integrated which can accurately move a burr along a computer-generated trajectory. The workspace of the robot is specifically adapted for unicompartmental knee arthroplasty considering inherent safety. The objective is to provide a versatile robotic tool which improves the accuracy of implant positioning and reduces the revision rate, as reported in literature [17, 19, 20], while optimizing its integration into the conventional surgical workflow.

The requirements of the robot developed were defined as a lightweight (< 3 kg) and handy design with three degrees of freedom for positioning a standard surgical milling tool. Bast et al. measured the maximum forces during bone milling to be 21 N [21]. With a safety factor of at least two, the robot developed must be capable of withstanding forces of 45 N.

A dynamic motion control should be able to compensate for the movements of the surgeon and of the bone. Therefore, the system must, at least, be able to correct the surgeon’s tremor and drift, the former being at frequencies of 8–12 Hz [22].

In an animal study, Sandborn et al. found out that a gap of 0.5 mm or less between bone and implant enhances bone ingrowth [23]. Therefore, an accuracy of 0.5 mm should be achieved together with a feed rate of up to 10 mm/s to allow the efficient machining of bone.

The purpose of this paper is to characterize the control loop and evaluate whether the requirements for a dynamic motion compensation given can be met by a lightweight, handheld burring robot.

## System and controller description

The first subsection gives a brief overview of the manipulator. This is followed by a description of the system architecture and finally the control loop.

### Manipulator design

**Fig. 1:**
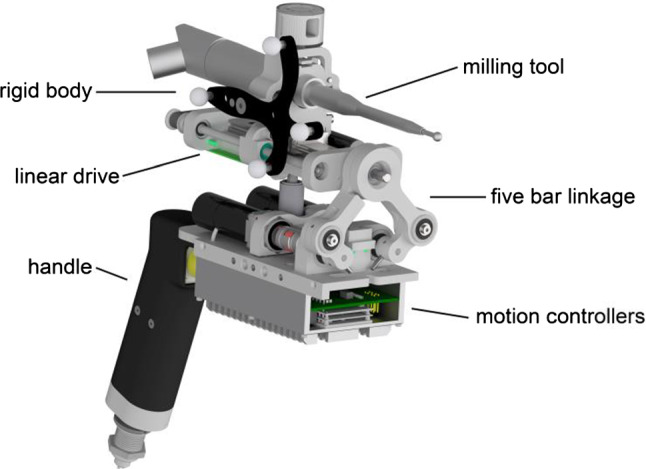
CAD rendering of the 3 DoF robot developed incorporating a standard high-speed milling tool and a rigid body for optical tracking

**Fig. 2:**
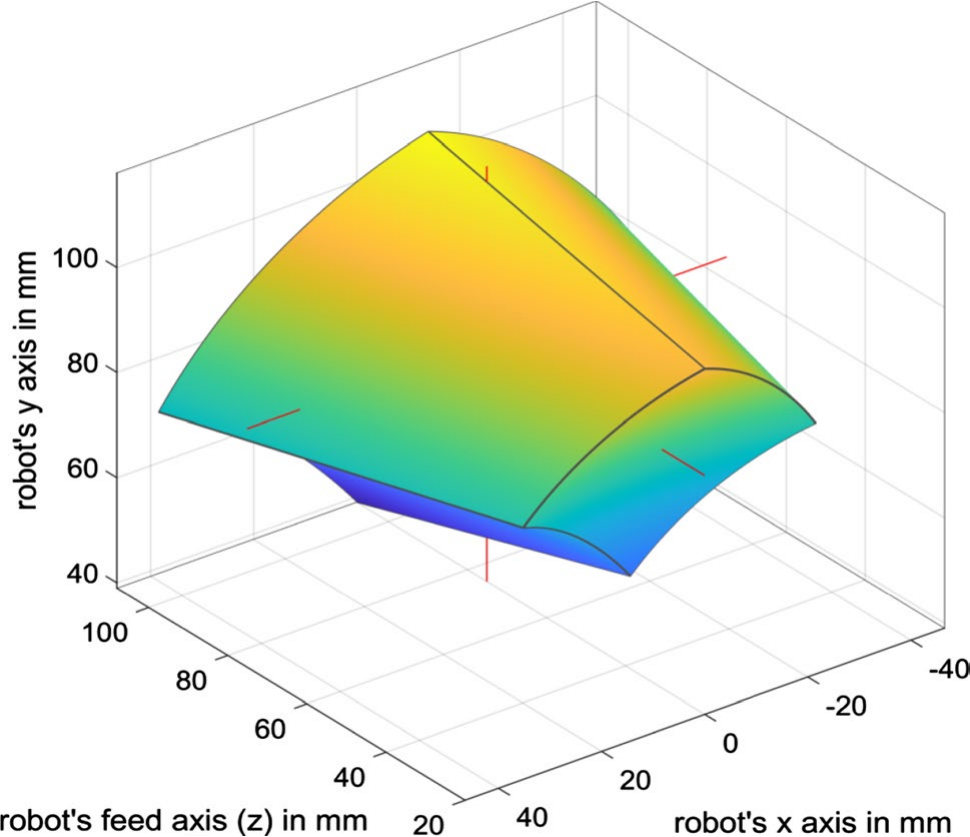
Workspace of the manipulator. The red lines point towards the center of the workspace, being at $(0, 84.4, 75.1) \,\mathrm{mm}$. The center of the workspace is defined as the center of the biggest sphere fitting inside the hull, having a radius of $25 \,\mathrm{mm}$

A miniaturized robot with a total weight of 2.5 kg, including power tool and cables, was designed (Fig. 1), based on a five-bar linkage mechanism and a linear drive. The workspace of this manipulator is shown in Fig. 2.

A standard surgical high-speed burr is mounted onto the end of the kinematic chain together with a rigid body for the localization of the tool. The position of the burring tip in relation to the rigid body is calibrated prior to each intervention since the clamping of the tool onto the draped robot cannot be reproduced perfectly. Using a spherical rosen burr, the burr axis for the unicompartmental knee arthroplasty procedure can be chosen freely by the surgeon within the limits of the surgical approach and does not need to follow a specific plan. Therefore, three DOF of the robot are sufficient to maneuver the tool along a preplanned trajectory.

The user can hold and position the robot using a single-hand grip at the rear end, which also hosts a button to activate the burr. The second hand can be placed on the patient’s lower leg while stabilizing and supporting the front end of the robot.

After the manipulator has been pre-positioned and activated by the surgeon, the burring tool is automatically moved by the robotic system along a previously planned trajectory. In case the burring tool cannot be moved further at one position, as it would then exceed the manipulator’s workspace, the burring tool is stopped by the system and the surgeon is informed to re-align the manipulator.

The entire procedure is closely monitored by the surgeon. In case of any unforeseen event, the manipulator can be stopped at any time or, since it is a handheld device, just being withdrawn from the surgical site by the operator.

### System architecture

**Fig. 3:**
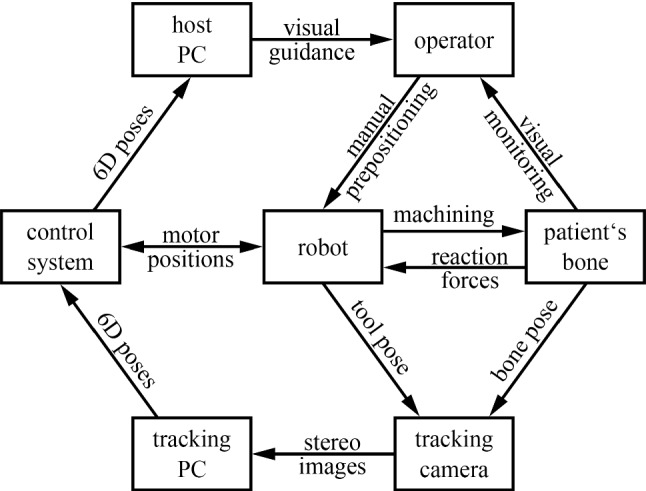
Simplified architecture of the robotic system; arrows indicate the flow of information and forces

The centerpiece of the MINARO HD robotic system (Fig. 3) is a real-time control system development platform with a DS1006 processor board (dSPACE GmbH, Paderborn, Germany). It is equipped with several expansion cards for CAN and serial communication, analog and digital in- and outputs, and host PC communication.

The robotic manipulator is equipped with three motion controllers that communicate with the control system via the CiA 402 profile (CAN in Automation) with a 3 ms cycle time. Position and velocity demand values are sent to the manipulator from the control system, whereas the motion controllers return actual position, velocity and current measurements.

The burring tool mounted on the manipulator can be activated by the dSpace control system. It is set to a fixed rotational speed of 60,000 rpm. The current drawn by the burring tool can be measured with an added shunt resistor through the dSpace DS2004 High-Speed A/D expansion board. Thus, the relative course of the machining force can be monitored.

The patient is simulated by a fixture for a synthetic bone substitute material and a rigid body localized by the tracking camera. An FTS-Mini-45 force torque sensor (SCHUNK GmbH & Co. KG, Lauffen/Neckar, Germany) is mounted between the bone phantom and the rigid fixture to measure the machining forces directly. The sensor is read out by the DS2004 card of the dSpace system.

Both the bone phantom and the robot’s tool are tracked by a fusionTrack 500 tracking camera (Atracsys LLC, Puidoux, Switzerland) with an update interval of 3 ms. The compressed stereo images are sent through a Gigabit Ethernet connection to a dedicated tracking PC running on Windows Embedded Compact. The atracsys software development kit is used to compute the 6D poses of both rigid bodies. Those poses are then sent to the control system via an RS 422 connection running at 1.2 MBaud. A direct connection of the tracking camera to the dSpace real-time system is not possible because the atracsys software development kit cannot be compiled for this platform.

A host PC is connected to the control system via an optical link and shares information with the running real-time control using the ASAM (Association for Standardization of Automation and Measuring Systems) standard XIL API (model/software/processor/hardware-in-the-loop application programming interface). The software of the host PC guides the user through the intervention, from homing all axes of the robot, registering the patient dummy and uploading a trajectory to the real-time control prior to the intervention, up to showing a compensatory navigational display for the rough prepositioning of the manipulator by the user. The user is guided through the different steps of the intervention by using a medical multifunction foot switch (steute Technologies GmbH & Co. KG, Löhne, Germany).

### Control architecture

**Fig. 4:**
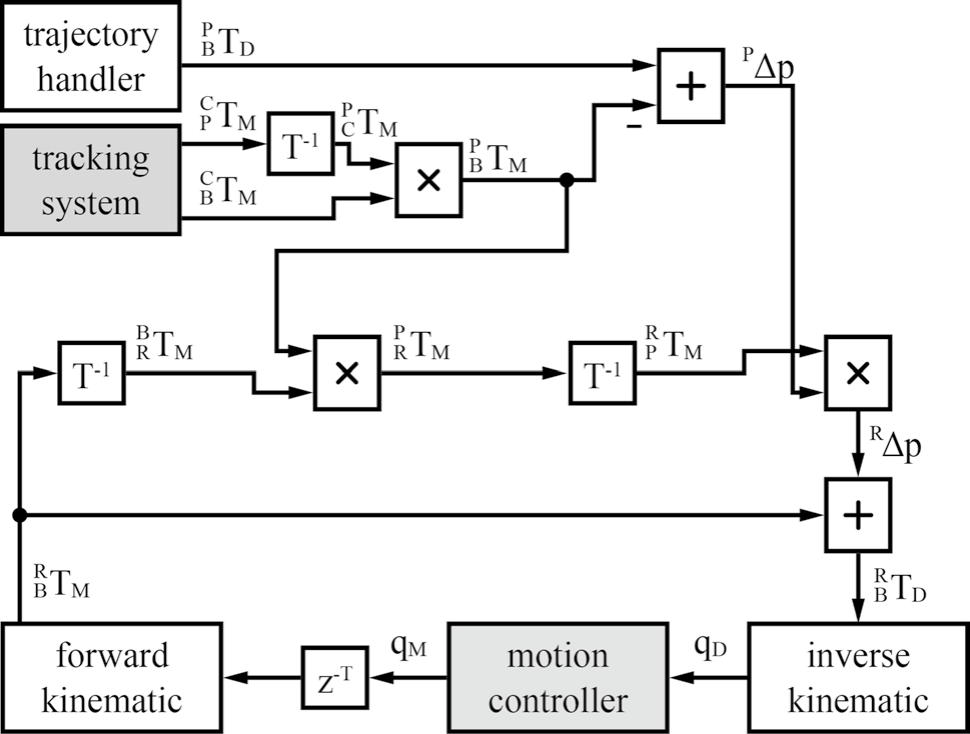
Control loop to compute the target joint positions *q*_D_. The tracking system and motion controllers provide the sensor data, whereas all calculations (all white blocks) are performed on the dSpace control system. The joint positions required are then sent to the motion controllers

The objective of the outer control loop, running on the real-time dSpace platform, is a dynamic compensation for introduced disturbance movements by the user and the patient, while following a planned milling path. Therefore, the positioning error of the burring tool ${}^{P}\Delta p$ is constantly monitored by the tracking camera and deviations are compensated in a control loop similar to [14], as can be seen in Fig. 4.

Forward and inverse kinematics must be calculated for the control loop. An analytical solution using the geometric dimensions derived from the CAD model has been implemented for both transformations, i.e., from the robot base’s coordinate system to the tool’s coordinate system and vice versa. The origin of the robot base’s right-handed coordinate system is centered between the motor axes of both lower motors driving the five-bar linkage and in the plane that also goes through the center of the ball joint in the coupling point. The y vector of the coordinate system points upwards, perpendicular to the robot’s base plate. The z vector points frontwards, towards the patient.

The origin of the tool’s right-handed coordinate system is in the center of the burring tool. The z vector points towards the patient, in the direction of the tool’s shaft. The y vector also points upwards, perpendicular to the linear guide.

The actual joint positions, measured by the motion controllers, are transmitted via CANopen. Since measurement and transmission of the joint positions is faster than the tracking camera, an extra delay must be added to the joint positions such that both measurements originate from the same real event. The exact duration of the delay required will be measured during the following experiments.

The transformation ${}_{B}{}^{R}{T}_{M}$ from the robot’s base *R* to the burring tool* B* is calculated with the direct kinematic model of the manipulator and the measured joint positions ${q}_{M}$. In parallel, the tracking camera measures the patient’s pose *P* as ${}_{P}{}^{C}{T}_{M}$ and the burring tool pose ${}_{B}{}^{C}{T}_{M}$ in its own coordinate system *C*. The former is inverted and multiplied with the latter to receive the burring tool’s pose in the patient’s reference frame ${}_{B}{}^{P}{T}_{M}$. Multiplying this transformation with the known robot base pose in the burring tool’s frame ${}_{R}{}^{B}{T}_{M}$ from the encoder position leads to the robot base pose in the patient’s coordinate system ${}_{R}{}^{P}{T}_{M}$, which is inverted to ${}_{P}{}^{R}{T}_{M}$.

From the transformation ${}_{B}{}^{P}{T}_{M}$ of the current burring tool pose in the patient’s coordinate system, the translational part is taken and subtracted from the desired position ${}_{B}{}^{P}{T}_{D}$, resulting in the distance vector ${}^{P}\Delta p$, resembling the current position error in the patient’s coordinate system.

This error vector is multiplied to the rotational part of the transformation ${}_{P}{}^{R}{T}_{M}$. This results in the vector ${}^{R}\Delta p$, which is the error vector in the robot’s base coordinate system. This translational vector is added to the measured transformation ${}_{B}{}^{R}{T}_{M}$ of the burring tool in the robot’s base coordinate system, resulting in the desired position ${}_{B}{}^{R}{T}_{D}$ of the burring tool in the robot’s base coordinate system. After applying the inverse kinematic model of the manipulator, the desired joint positions ${q}_{D}$ are ready to be sent to the motion controllers.

This outer loop is executed on the dSpace system every millisecond, even though an update from the motion controllers and the tracking camera is only received every 3 ms. The trajectory handler updates the burring tool’s required position every millisecond, following the trajectory that was programmed into the control system by the host PC. The feed rate of the burr on the planned trajectory can be defined by the user.

The three motion controllers on the manipulator are operated in the profile position mode. The position controller computes a position trajectory from the actual and the joint position required, taking into account the maximum acceleration defined, deceleration and speed of the motors. Using cascaded PI controllers, consisting of a position controller, velocity controller and current controller, the motion controller follows this trajectory until a new joint position requirement is received from the control running on the dSpace system.

## Evaluation method

The control has been evaluated to verify whether the requirements regarding the dynamic motion compensation are met by the robotic system. The components tracking system and motion controllers are tested separately. Regarding the tracking system, the latency between a change in the real world and the arrival of this information at the dSpace control system is measured. Regarding the manipulator, a position step is given in Cartesian coordinates and the response of the motors is recorded.

**Fig. 5:**
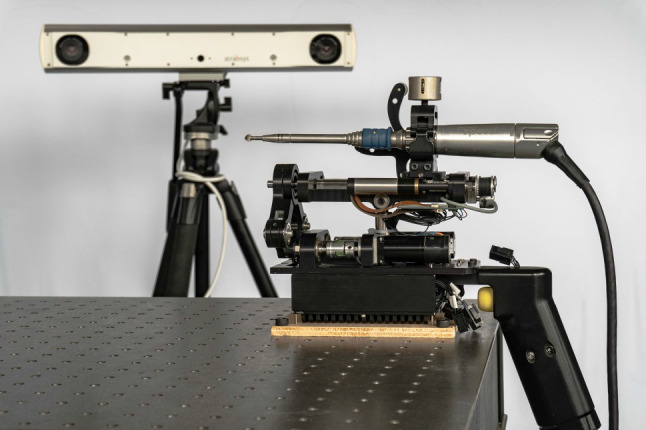
Photograph of the test setup consisting of Minaro HD and tracking camera. For this solely technical evaluation of the robot dynamic, the otherwise handheld robot was rigidly fixed to a table

Afterwards, the complete control is evaluated. Firstly, the step response is recorded again but this time, the step is given in the patient coordinate system and the response is recorded using the tracking camera. Using this test, the maximum frequency of disturbing movement that can be compensated for by the robot is evaluated. Secondly, the positioning error of the robot depending on the velocity of the disturbing movement or the feed rate of the burring process required is measured. A photography of the experimental test setup can be seen in Fig. 5.

### Tracking: latency

A single infrared (IR) LED connected to the dSpace control system is used to measure the latency of the tracking system. After the IR LED is turned on by the dSpace system, it is recognized as a stray fiducial by the tracking camera. The dedicated tracking PC processes the information received from the camera and sends the location of the IR LED to the dSpace system. The dSpace system measures the time between turning on the IR LED and receiving its location from the tracking system. Subsequently, the dSpace system turns off the IR LED and measures the latency again, this time until the LED location is no longer received from the tracking system.

### Motion controller: step response

Only the lower row of the diagram in Fig. 4 is evaluated to test the motion controllers separately. Cartesian steps of 2 mm are applied to the target tool position ${}_{B}{}^{R}{T}_{D}$ and the actual tool position ${}_{B}{}^{R}{T}_{M}$ is recorded. One step is applied in the z direction of the manipulator (“forward,” feed axis) that mainly requires the spindle drive to move. A second step is applied in the x direction (“sideways”) that mainly requires the two motors of the five-bar linkage to move. The third and last step is applied in a diagonal direction.

The three-dimensional movement of the tool position, measured by the joint positions and calculated using the forward kinematic, is projected to a one-dimensional movement along the direction of the step. From this, the latency, overshoot, rise time and settling time are extracted. In this context, latency is the time until the tool position movement passes the 5% mark, i.e., 0.1 mm. Overshoot will be given in percent and the rise time is the time required to rise from 5 to 95%, i.e., from 0.1 to 1.9 mm. The error window for the settling time is 10%, i.e., ± 0.2 mm. This is set as the accuracy required of the manipulator itself, so that some safety cushion remains, e.g., for tracking inaccuracies and movements due to reaction forces by the burring tool. To evaluate the maximum frequency f of the disturbing movement that can be compensated for by the robot, the 0 to 90% rise time will be read off and be called the reaching time *T*. The maximum frequency *f* then equals $1/(2T)$.

This and all subsequent measurements are only taken once. Due to the repeatability of robotic behavior, this is assumed to be sufficient.

### Control loop: step response

The step response of the whole control loop is evaluated analogously to the step response of the manipulator. Only the step is applied to the target tool position ${}_{B}{}^{P}{T}_{D}$ in the patient’s coordinate system and the actual tool position ${}_{B}{}^{P}{T}_{M}$ in the patient’s coordinate system, recorded by the tracking camera, is taken as the response. Since the manufacture and calibration of the manipulator is not perfect, the joint positions required are updated with every tracking frame, converging to the burr position needed.

### Positioning error versus disturbing movement speed

Regarding these experiments, disturbing movements of 50 mm amplitude and with various speeds and directions are added to the location ${}_{P}{}^{C}{T}_{M}$ of the patient. The movements are applied in the same directions as the step responses and the speeds range from 1 up to 50 mm/s. The maximum positioning error ${}^{P}\Delta p$ is recorded during each test run.

## Results

### Tracking: latency

The latency between a change in the real world and the corresponding data reaching the dSpace control system could be measured between 6 and 8 ms, with rare dropouts of up to 16 ms.

Since the reception of the manipulator joint positions via the CANopen bus takes between 2 and 4 ms until it is processed by the dSpace system, the extra latency ${z}^{-T}$ of the control loop (seen in Fig. 4) is set to 4 ms for all following experiments.

### Motion controller: step response

**Fig. 6:**
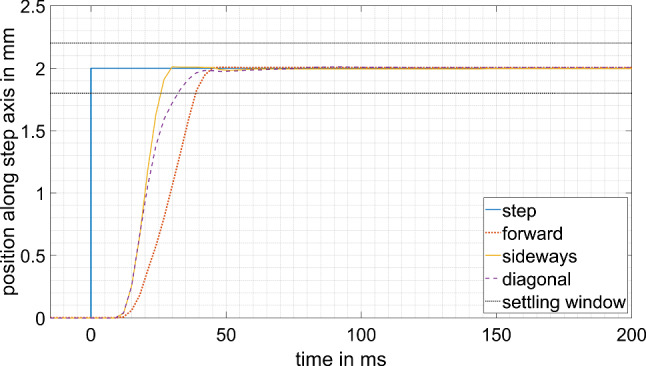
Step response of the motion controllers

The step response measurements of the manipulator alone can be seen in Fig. 6; the evaluation is summarized in Table 1. The reaching time (sum of the latency and rise time) of the step responses varies between 27 and 39 ms, depending on the direction of the step. This results in a maximum compensation frequency of 13–19 Hz.

**Table 1:** Evaluation of the motion controller step response

	Forward	Sideways	Diagonal
5% latency (ms)	18	15	15
Overshoot (%)	0.4	0.6	0.6
5–95% rise time (ms)	24	12	21
± 10% settling time (ms)	39	27	33
90% reaching time (ms)	39	27	33

### Control loop: step response

**Fig. 7:**
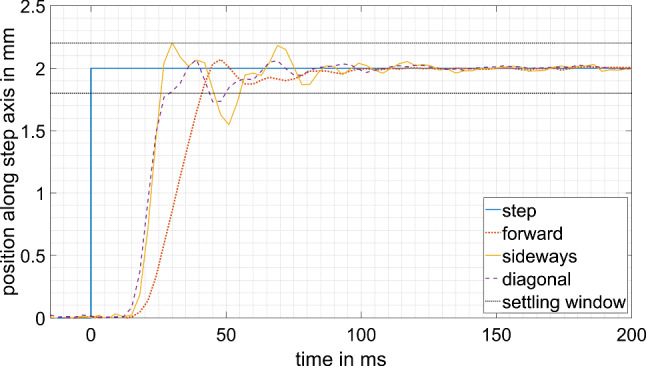
Step response of the control loop

The step response measurements with the tracking camera in the control loop can be seen in Fig. 7; the evaluation is summarized in Table 2. The reaching time varies between 27 and 42 ms, resulting in a maximum compensation frequency of 12–19 Hz.

**Table 2:** Evaluation of the control loop step response

	Forward	Sideways	Diagonal
5% latency (ms)	21	18	18
Overshoot (%)	3.5	10.0	3.6
5–95% rise time (ms)	24	9	18
± 10% settling time (ms)	42	57	51
90% reaching time (ms)	42	27	30

### Positioning error against disturbing movement speed

**Fig. 8:**
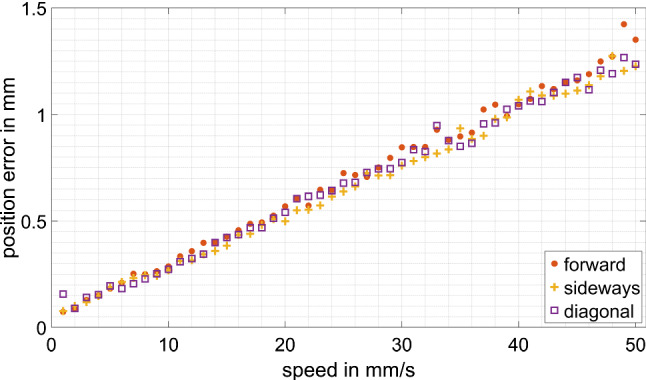
Positioning error against disturbing movement speed

The positioning error (Fig. 8) is mostly linear with no distinct variation depending on the direction of the disturbing movement. The slope of a linear fit to the three curves is $0.025 \frac{\mathrm{mm}}{\mathrm{mm}/\mathrm{s}}=25\, \mathrm{ms}$ with 0.048 mm error offset.

A positioning accuracy of 0.5 mm can be achieved with relative movements between the patient and burring tool of up to 18 mm/s.

## Discussion

The dropouts of the tracking latency measurements can be explained by the tracking PC not running a hard, real-time operating system. The input of the control loop should be made more reliable by switching the operating system, for example, to real-time Linux.

The step responses show a significant latency until a movement of the joints is measured. This is due to the rather slow update rate of the CAN bus. Not only is the joint position currently exchanged between the control system and the motion controllers but also information, such as velocity and current, is transmitted for better understanding and development of the robotic system. The data amount can be reduced for later use and, therefore, the update rate could be increased while maintaining the baud rate of the bus. This would result in a lower latency of the motion controllers and, thus, shorter settling and reaching times, allowing for the compensation of higher frequency disturbance movements, such as reaction forces of the burring process. Nevertheless, the result of a maximum compensation frequency of 12–19 Hz already fulfills the given requirement of filtering the surgeon’s tremor at 8–12 Hz [22].

Comparing the step responses of the manipulator itself to the step responses of the whole control loop, no big differences of latency and rise time stand out. The slightly higher latency of the control loop can be explained by the tracking camera having 4 ms more latency than the joint encoder readout. The partially longer rise time of the motion controller step response might be due to a less aggressive velocity curve of the motion controllers. The acceleration and target velocity are calculated by the motion controllers, depending on the positioning error (suddenly changing to 2 mm with the step input). The demand position while testing the control loop might have changed by more than 2 mm due to inaccuracies between the kinematic model and the real manipulator, thus, resulting in higher target acceleration and velocity of the motion controller to reach this position.

This behavior could also explain the overshoots during the step response measurement of the control loop. Since there is basically no overshoot in the step response of the manipulator itself, the motion controllers are well tuned to stop exactly at the joint position required. However, the initial target joint position sent to the motion controllers might have been too high due to imperfect calibration of the kinematic model. The control recognizes the wrong calculation while approaching the target position of the 2 mm step in the patient’s coordinate system and adjusts the joint demand positions. The tool position overshoots due to the latency in the control.

The 25 ms slope of the positioning error against the disturbing movement speed is only slightly higher than the latency of the control loop. It should equal the reaching time of the control loop for very small step sizes. The 48 µm error offset can be explained by the noise of the tracking camera. The linearity of the taken measurements, with no outliers, shows that the assumption regarding repeatability of the robotic system was correct and therefore a sample size of one measurement per setting was sufficient.

In summary, the dynamic behavior of a new, handheld robotic device for burring tasks, like bone removal for unicompartmental knee arthroplasty, was analyzed. The step response of the robotic system was measured as well as the capability to follow a moving target. Additionally, the design of the manipulator was presented, as well as the system and control architecture. The results show that the requirements regarding compensation frequency, accuracy and feed rate are met by the novel handheld MINARO HD robot. With a maximum feed rate required of 10 mm/s and a 0.5 mm accuracy achieved for relative movements of up to 18 mm/s, the MINARO HD will be able to compensate simultaneously for unintended movements of the operator or the patient while maintaining the trajectory path and feed rate required.

To the best of the author’s knowledge, this is the smallest robot for surgical burring tasks that can autonomously move the burr along a previously generated trajectory while adapting the milling parameters to prevent excessive temperature rises and milling forces while achieving high surface flatness [18].
